# Design of Friction Stir Spot Welding Tools by Using a Novel Thermal-Mechanical Approach

**DOI:** 10.3390/ma9080677

**Published:** 2016-08-09

**Authors:** Zheng-Ming Su, Qi-Hong Qiu, Pai-Chen Lin

**Affiliations:** Advanced Institute for Manufacturing with High-Tech Innovations & Mechanical Engineering, National Chung-Cheng University, Chia-Yi 621, Taiwan; suchm1011@gmail.com (Z.-M.S.); new7100222000@yahoo.com.tw (Q.-H.Q.)

**Keywords:** tool design, thermal-mechanical model, friction stir spot welding, spot friction welding, lap-shear specimen

## Abstract

A simple thermal-mechanical model for friction stir spot welding (FSSW) was developed to obtain similar weld performance for different weld tools. Use of the thermal-mechanical model and a combined approach enabled the design of weld tools for various sizes but similar qualities. Three weld tools for weld radii of 4, 5, and 6 mm were made to join 6061-T6 aluminum sheets. Performance evaluations of the three weld tools compared fracture behavior, microstructure, micro-hardness distribution, and welding temperature of welds in lap-shear specimens. For welds made by the three weld tools under identical processing conditions, failure loads were approximately proportional to tool size. Failure modes, microstructures, and micro-hardness distributions were similar. Welding temperatures correlated with frictional heat generation rate densities. Because the three weld tools sufficiently met all design objectives, the proposed approach is considered a simple and feasible guideline for preliminary tool design.

## 1. Introduction

Resistance spot welding is widely used to join body-in-white parts made of steel sheets in the automotive industry. However, resistance spot welding of aluminum sheets likely produces unsatisfactory welds due to numerous voids and defects inside the nugget. Iwashita [1] developed a new spot joining process, evolved from friction stir welding (FSW), to join aluminum sheets. This new process was denoted as friction stir spot welding (FSSW) and could make joints without melting the base metal (BM). Consequently, the voids and defects from rapid solidification of fused aluminum alloy can be easily avoided.

In order to widely apply the FSSW process to the automotive industry, a simple guideline or approach for tool design could be very helpful. In order to develop a tool design approach, the effects of tool size and shape on the mechanical properties of FSSWs should be determined. Tozaki et al. [2] demonstrated how probe length affects FSSWs in aluminum 6061-T4 lap-shear specimens. Tozaki et al. [3] showed how shoulder radius affects those welds. Their experimental results indicated that the mechanical properties of FSSWs depend mainly on probe length but also slightly on shoulder radius. Lin et al. [4,5] investigated how shoulder geometry affects FSSWs in aluminum 6111-T4 lap-shear specimens. Their experimental results confirmed that the fracture and fatigue behaviors of FSSWs made by flat and concave weld tools are determined by microstructures and weld geometries. Badarinarayan et al. [6] investigated how probe geometry affects FSSWs in aluminum 5754-O lap-shear specimens. They found that, compared to welds made by circular probes, those made by triangular probes are of superior quality, possibly due to additional back-and-forth force. Notably, the “triangular” here indicates that the cross section of a probe has a triangular shape with rounded corners.

For a given weld radius, the shoulder radius and probe length of weld tools can be easily determined based on the results of Tozaki et al. [2]. Notably, the size of FSSWs here is defined as the shoulder indentation radius. However, no rule or guideline is available for determining an appropriate probe radius. Tozaki et al. [3] indicated that an inappropriate tool design can lead to unsatisfactory mechanical properties for FSSWs under most processing conditions. It should be noted that the rotating tool shoulder and probe in the FSSW process generate frictional heat to soften sheet metals adjacent to the weld tool. The probe then stirs the softened materials to make a solid-state bond between the upper and lower sheets. Therefore, tool dimensions significantly affect both heat generation and material flow during welding. Tozaki et al. [2] indicated that tool dimensions also affect the microstructure and failure load of FSSWs. Therefore, a thermal-mechanical model for FSSW, which correlates frictional heat and material flow with tool dimensions, is needed to provide guidelines for tool design.

This work presents a simple thermal-mechanical model for FSSW that correlates tool dimensions with tool performance. The thermal-mechanical model associated with a combined approach enables design of weld tools for welds of various sizes but similar qualities. Three weld tools for weld radii of 4, 5, and 6 mm are made to join 6061-T6 aluminum sheets. Finally, performances of the three weld tools are then evaluated in terms of microstructure, failure load, micro-hardness, and welding temperature of welds in lap-shear specimens.

## 2. Thermal-Mechanical Model for FSSW

**Figure 1:**
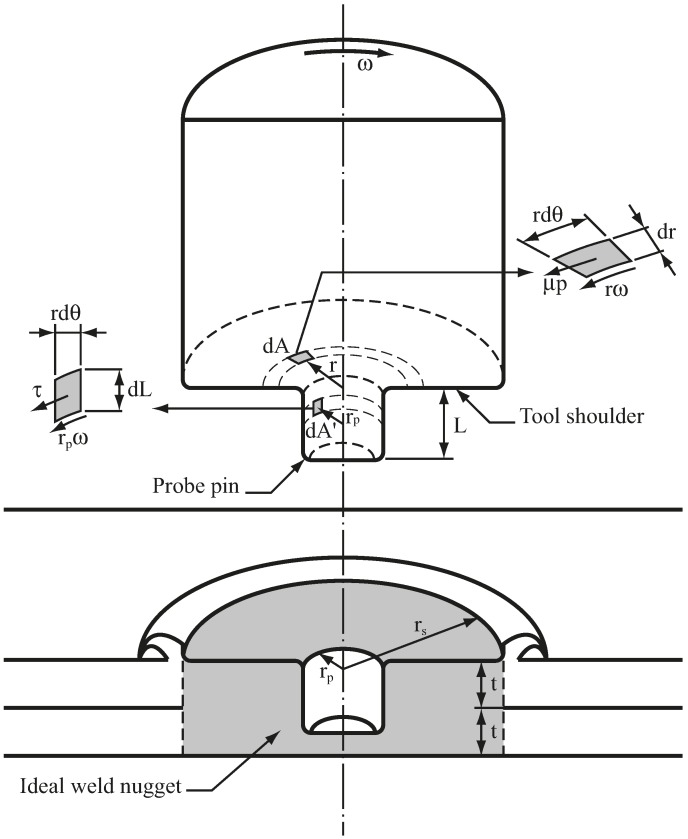
Schematic plots of the halves of an extracted weld tool and an ideal FSSW.

Chao and Qi [7] indicated that FSW has two main heat sources, friction and plastic deformation. The friction generates heat from the interface between the tool surface and neighboring materials. The plastic deformation generates heat from the weld materials deformed and stirred by the rotating tool. They also indicated that the frictional heat is the main heat source of the FSW process. Therefore, only the frictional heat will be considered in the following. The thermal-mechanical model for FSW in Colegrove [8] showed that frictional heat generation correlates with tool dimensions and processing parameters. Although FSW and FSSW have different material flow and transport characteristics, the two processes have similar mechanisms of frictional heat generation. Based on the approach of Colegrove [8], the approximate heat generation rate of a weld tool $\dot{Q}$ is given as

(1) $$\dot{Q}=\int_{r_{p}}^{r_{s}}r\omega \left(\mu p2\pi rdr\right)+\int_{0}^{L_{p}}r_{p}\omega \left(C_{f}\mu p2\pi r_{p}dL\right)+\int_{0}^{r_{p}}r\omega \left(\mu p2\pi rdr\right)=\frac{2\pi \mu p\omega r_{s}^{3}}{3}+2\pi C_{f}\mu p\omega r_{p}^{2}L_{p}$$

where μ is the friction coefficient; p is the average pressure over the weld tool; ω is the angular speed of the weld tool; dA is a differential area of the weld tool; $r_{s}$ is the shoulder radius; $r_{p}$ is the probe radius; and $L_{p}$ is the probe length (Figure 1). The $C_{f}$ is a geometric factor to account for the contact area along the probe circumference. In Equation (1), the first integral represents the frictional heat generation rate from the tool shoulder and the second and third integrals represent those from the probe. Note that without further information, μ is assumed to be a constant. For a threaded probe with a thread angle of 60°, $C_{f}$ is defined as 2.0 since the contact area is almost double that of a smooth probe.

Based on the similar approach, the approximate stirring power/capacity of a probe P is derived as

(2) $$dP=r_{p}\omega \left(C_{s}\tau dA\prime \right)\;P=\int_{0}^{L_{p}}r_{p}\omega \left(C_{s}\tau 2\pi r_{p}dL\right)=2\pi C_{s}\mu \tau \omega r_{p}^{2}L_{p}$$

where τ is the shear stress for stirring motion and dA′ is a differential area of the probe (Figure 1). Here, τ is assumed to be a constant; the $C_{s}$ is a geometric factor, which represents the stirring capacity from features on a probe; and for a threaded probe, $C_{s}$ is defined as 1.0 since this is the simplest and most commonly used feature. If further evidence supports a different $C_{s}$ value, this approach remains applicable. Welding performance can then be evaluated by defining parameters based on the geometric parameters of Equations (1) and (2), respectively. The two parameters, α and β, are given as

(3) $$\alpha =\frac{2\pi r_{s}^{3}+6\pi C_{f}r_{p}^{2}L_{p}}{3W}$$

(4) $$\beta =\frac{2\pi C_{s}r_{p}^{2}L_{p}}{W}$$

and

(5) $$W=2\pi r_{s}^{2}t-\pi r_{p}^{2}L_{p}$$

where W is the volume of the weld nugget and t is the sheet thickness (Figure 1); Volume W must be considered when comparing welds/tools of various sizes; parameters α and β correlate with the heat generation rate density and stirring power/capacity density of a weld tool, respectively. For comparing welds/tools of various sizes, the welding performance here is defined as heat generation rate density and stirring power density instead of heat generation rate and stirring power. Therefore, these parameters can be used to estimate welding performance and to suggest appropriate tool dimensions.

## 3. Tool Design Process

Aluminum 6061-T6 sheets with a thickness of 1 mm were used for FSSW. Weld radii of 4, 5, and 6 mm were considered. Notably, the weld radius was taken as the shoulder radius $r_{s}$ here (Figure 1). The shoulder radii of the three weld tools were set to 4, 5, and 6 mm, respectively. Since total sheet thickness was 2 mm and maximum shoulder indentation depth was 0.3 mm, the probe length of the three weld tools should be $2t>\left(L_{p}+d_{max}\right)>t$ to avoid penetration during welding. The $d_{max}$ was the maximum shoulder indentation depth. Therefore, the probe length was set to 1.6 mm in Table 1. The thermal–mechanical model for FSSW was then used to determine the probe radii of the three weld tools.

To design weld tools for various sizes but similar qualities, identical α and β values were needed based on the thermal-mechanical model. These two parameters strongly correlated with welding performance. The initial tool, which had a shoulder radius of 5 mm, a probe radius of 2 mm, a probe length of 1.6 mm, and metric threads of M4, was used as a basis for designing the other two weld tools. This tool design apparently was not considered optimal. However, Lin et al. [9] and Tozaki et al. [2,10] showed that the weld tools with similar designs provide satisfactory welds in sheets with thicknesses of 1.0 and 2.0 mm. Additionally, the power limitations of the FSSW machine precluded a probe radius exceeding 2.5 mm. Therefore, this tool design (T2 tool in Table 1) was used as a reference tool for all analyses.

Table 1 shows that parameters α and β of the T2 tool, according to the thermal-mechanical model in Equations (3) and (4), are 2.50 and 0.29, respectively. Geometric factors $C_{f}$ in Equation (3) and $C_{s}$ in Equation (4) for the threaded probe are 2.0 and 1.0, respectively. Table 1 shows that, given identical α values, the β values of T1a and T3a tools substantially differ from the reference value. In T1a and T3a tools, the relative deviations in β, $\delta_{\beta }$ ($=\left(\beta -\beta_{T2}\right)/\beta_{T2}$), are 49% and −43%, respectively. In contrast, given identical β values, the α values of T1b and T3b tools also differ from the reference value. The relative errors in α, $\delta_{\alpha }$ ($=\left(\alpha -\alpha_{T2}\right)/\alpha_{T2}$), in T1b and T3b tools are −15% and 15%, respectively.

Since identical α values and identical β values could not occur simultaneously, a compromise solution for this two-objective problem was needed. Based on the concept of optimization, both relative errors $\delta_{\alpha }$ and $\delta_{\beta }$ could be minimized simultaneously by minimizing their Euclidean norm. The Euclidean norm of $\delta_{\alpha }$ and $\delta_{\beta }$ was defined as

(6) $$\delta =\sqrt{\delta_{\alpha }^{2}+k^{2}\delta_{\beta }^{2}}$$

where k is a weighting factor of $\delta_{\beta }$ to account for the sensitivity of the welding performance on β; without losing generality; k was set to 1 here. If additional evidences support another value of k, this approach could also be applied.

**Figure 2:**
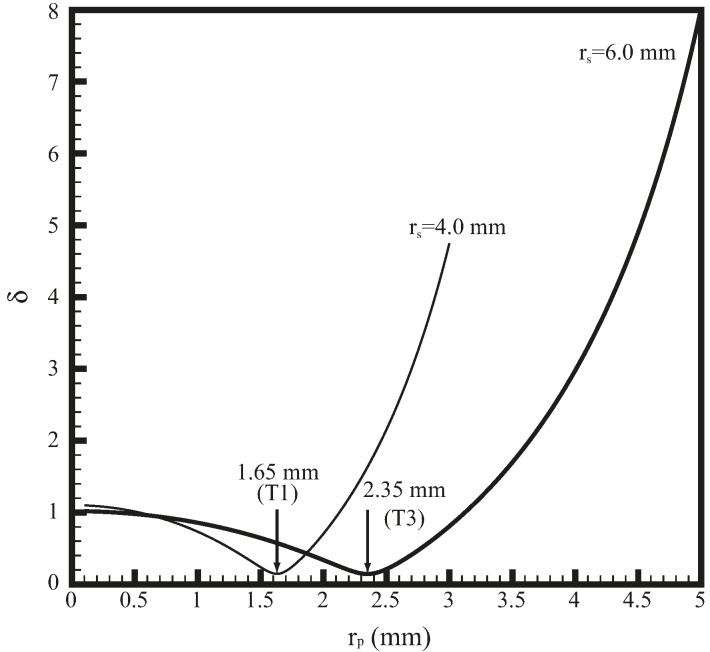
Euclidean norms δ as functions of probe radius $r_{p}$ and shoulder radius $r_{s}$.

Figure 2 shows the Euclidean norms δ as functions of probe radius $r_{p}$ and shoulder radius $r_{s}$. Sheet thickness t is 1 mm and probe length $L_{p}$ is 1.6 mm. As shown in Figure 2, the general trends of δ for different $r_{s}$’s are similar. Notably, $\delta_{\alpha }$ and $\delta_{\beta }$ are both minimized when δ is minimized. Therefore, T1 tool ($r_{s}=4\;\text{mm}$) and T3 tool ($r_{s}=6\;\text{mm}$) have probe radii of 1.65 and 2.35 mm, respectively. Table 1 shows that $\delta_{\alpha }$ of T1 and T3 tools decreases slightly, while $\delta_{\beta }$ of them decreases significantly. The $\delta_{\alpha }$ and $\delta_{\beta }$ for T1 tool are −13% and 7%, respectively, and those for T3 tool are 14% and −5%, respectively. Therefore, T1 and T3 tools are the most similar designs compared to T2 tool in terms of α and β.

## 4. Experiments

Aluminum 6061-T6 sheets with a thickness of 1 mm were used for FSSW. Table 2 lists the chemical compositions of the aluminum 6061-T6 sheets. Lap-shear specimens were made by using two 25.4 mm by 101.6 mm sheets with a 25.4 mm by 25.4 mm overlap area. The welds were made with a 5 hp numerically controlled milling machine under displacement-controlled conditions. A constant indentation rate of 2 mm/s was applied for all cases. The rotational speed ranged from 400 to 2000 rpm; the indentation depth ranged from 1.5 to 1.9 mm; the dwelling time ranged from 1 to 15 s.

**Figure 3:**
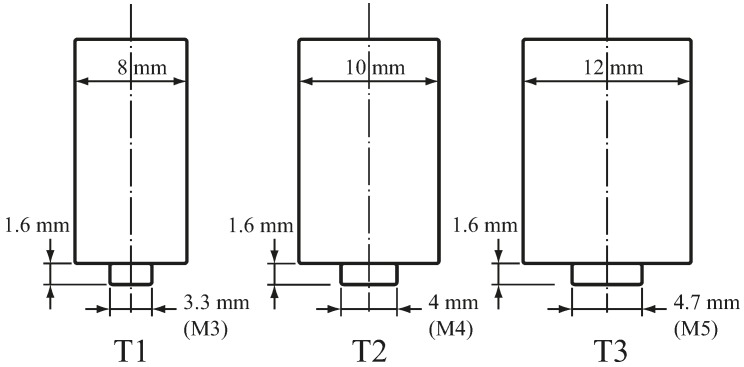
Schematic plots of three weld tools for weld radii of 4, 5, and 6 mm, denoted as T1, T2 and T3 tools, respectively.

**Figure 4:**
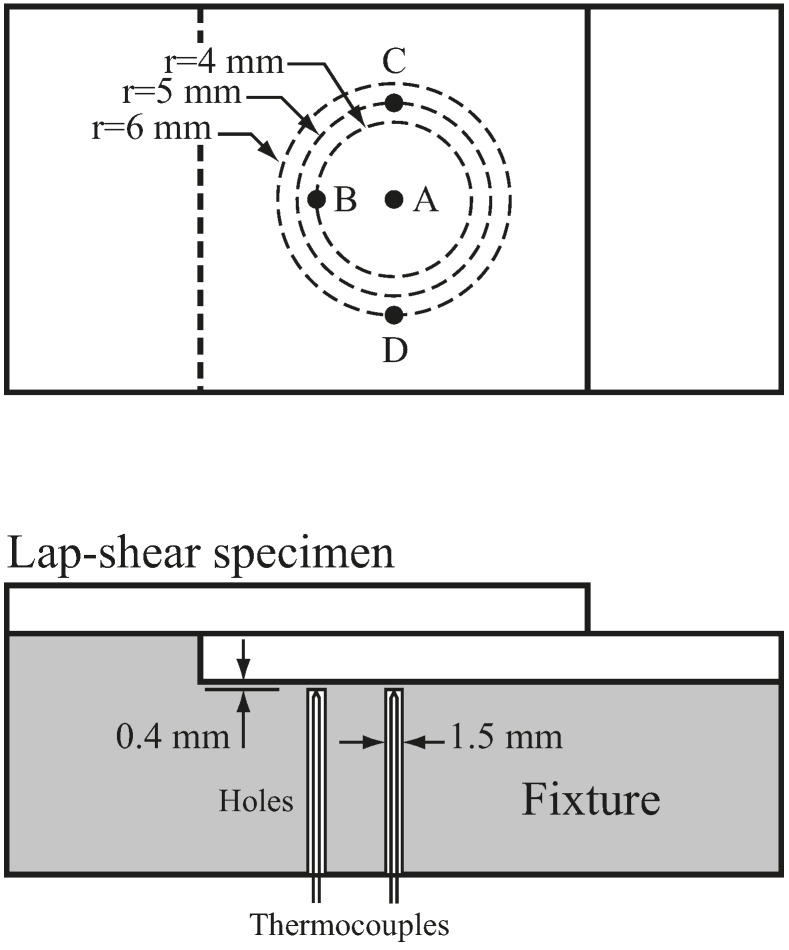
Schematic plots of thermocouples at locations A, B, C, and D in the fixture.

Three weld tools T1, T2, and T3 for weld radii of 4, 5, and 6 mm were used (Table 1). Figure 3 illustrates that the three weld tools have flat shoulders and threaded probes. Metric threads (M3, M4, and M5) with 60° thread angle were applied to the probes of the three weld tools. Notably, these threads were slightly modified to conform to their probe radii. The welding temperatures were measured by 0.08 mm diameter K-type thermocouples at locations A, B, C, and D in the fixture (Figure 4). The thermocouple has measurement accuracy of 0.1 Celsius degree and response time of 0.1 s. The tips of the thermocouples were installed 0.4 mm below the lap-shear specimen. Figure 4 shows location A is below the center of the nugget and locations B, C, and D are below the outer circumferences of T1, T2, and T3 tools, respectively. In Figure 3 and Figure 4, the three weld tools and fixture (anvil) were made of high speed steel SKD 11 and then heat treated.

**Figure 5:**
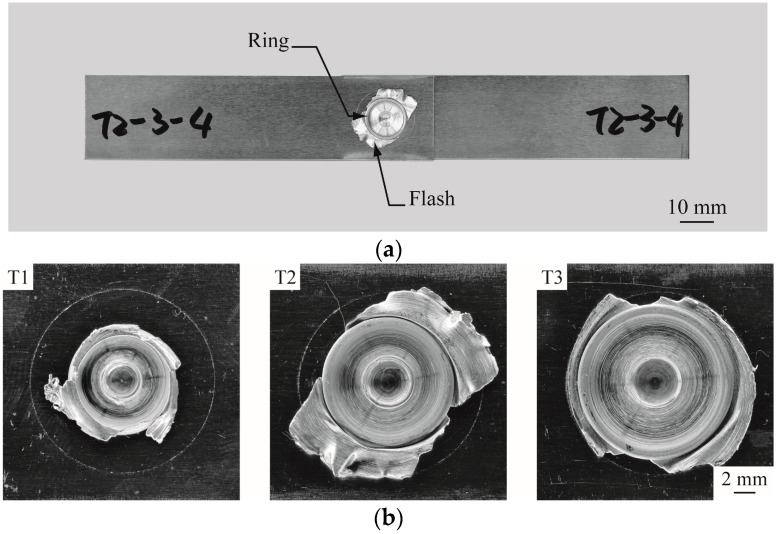
(**a**) A lap-shear specimen with a 6061-T6 FSSW made by T2 tool; (**b**) close-up top views of FSSWs made by T1, T2 and T3 tools, respectively.

Figure 5a shows a lap-shear specimen with a 6061-T6 FSSW made by T2 tool. The welds made by T1, T2 and T3 tools (Figure 5b) have a similar appearance and are slightly larger than the corresponding shoulder radii. Next, the performances of the three weld tools were studied by closely examining their weld qualities, including microstructure, failure load, failure mode, and micro-hardness distribution of welds in lap-shear specimens, as well as their welding temperatures. Notably, for each selected process condition, five to six specimens were made. At least three specimens were tested to obtain the average failure load. Two specimens were cross-sectioned to obtain the micrographs and the average TMAZ sizes.

## 5. Results

### 5.1. Micrographs of FSSWs before Testing

**Figure 6:**
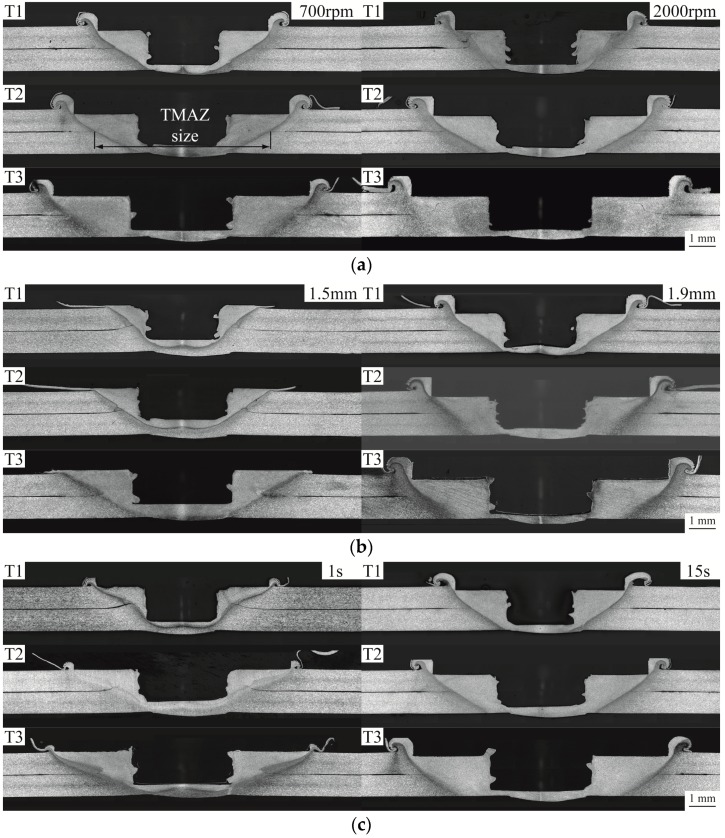
Optical micrographs of the cross sections of FSSWs made by the three weld tools at (**a**) rotational speeds of 700 and 2000 rpm; (**b**) indentation depths of 1.5 and 1.9 mm; and (**c**) dwelling times of 1 and 15 s.

**Figure 7:**
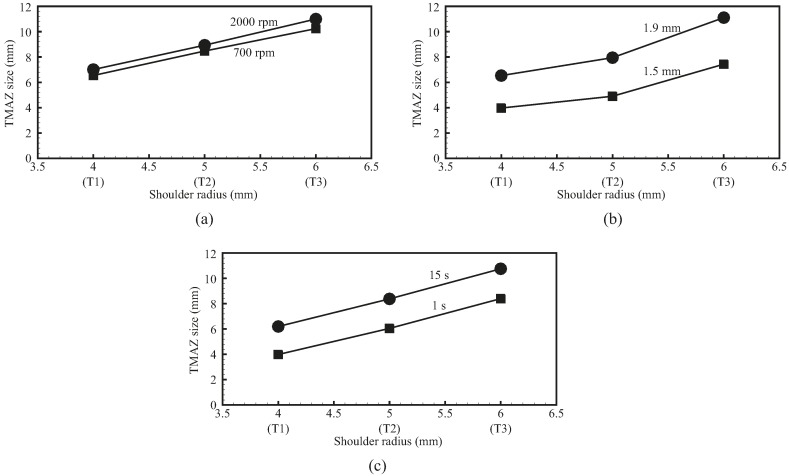
TMAZ sizes of FSSWs made by the three weld tools at (**a**) rotational speeds of 700 and 2000 rpm; (**b**) indentation depths of 1.5 and 1.9 mm; and (**c**) dwelling times of 1 and 15 s.

Figure 6 shows the optical micrographs of the cross sections of FSSWs made by the three weld tools at different rotational speeds, indentation depths, and dwelling times. Table 3 lists the processing parameters of the welds. As shown in Figure 6, the two gray areas near the central hole of welds are denoted as the thermal-mechanical affected zone (TMAZ) where the material is severely plastically deformed and recrystallized. Figure 7 shows the TMAZ sizes of FSSWs made by the three weld tools at various processing parameters. Notably, TMAZ size is defined as its width along the interfacial plane of the weld (Figure 6a). The TMAZ sizes in Figure 7 were measured from the micrographs in Figure 6. In addition, the TMAZ size is considered here since the failure of FSSWs in the circumferential failure mode is in general located near the boundary of TMAZ (Figure 9). This indicates that the TMAZ size has strong effects on the failure load as well.

Figure 6 and Figure 7 compare the welds made by the three weld tools. For the welds made by each weld tool under various processing conditions, the TMAZ sizes and profiles are substantially affected by indentation depth and dwelling time but slightly affected by rotational speed. For the welds made by the three weld tools under identical processing conditions, the TMAZ sizes are generally proportional to tool size and the TMAZ profiles are quite similar.

### 5.2. Failure Loads and Failure Modes of FSSWs

**Figure 8:**
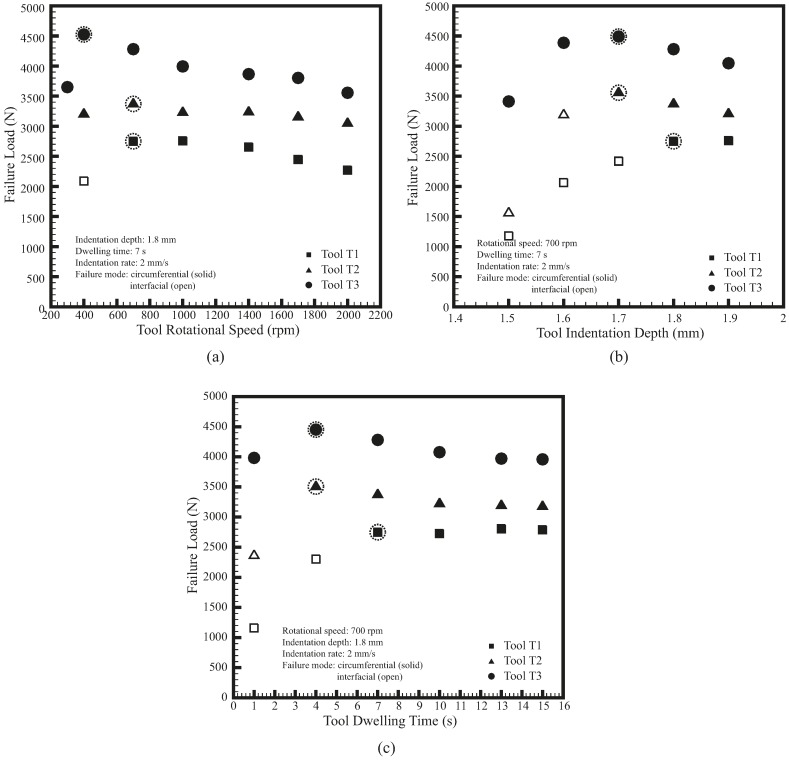
Average failure loads as functions of (**a**) rotational speed; (**b**) indentation depth; and (**c**) dwelling time for FSSWs made by the three weld tools under lap-shear loading conditions.

**Figure 9:**
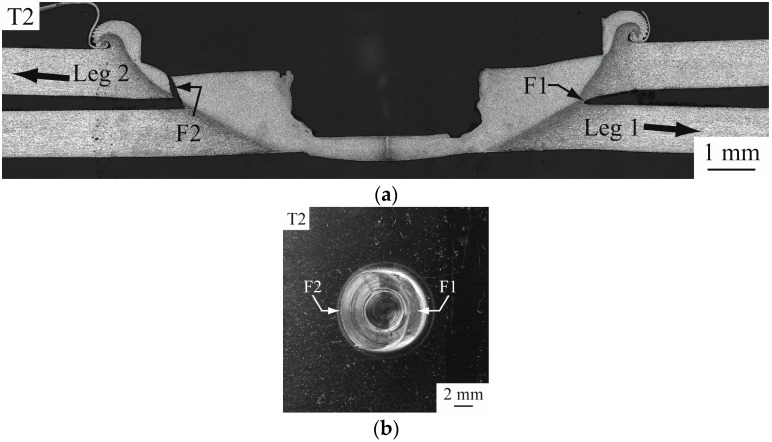
(**a**) An optical micrograph of the cross section of a partially failed FSSW; (**b**) A top view of another failed FSSW on the lower sheet.

**Figure 10:**
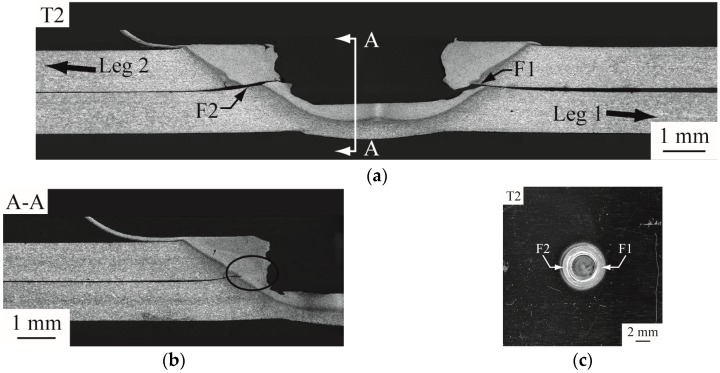
(**a**) an optical micrograph of the cross section of a partially failed FSSW; (**b**) an optical micrograph of the cross section along A-A plane in (**a**); (**c**) a top view of another failed FSSW on the lower sheet.

Figure 8 shows the average failure loads as functions of rotational speed, indentation depth, and dwelling time for FSSWs made by the three weld tools under lap-shear loading conditions. The solid symbol represents the nugget pullout or circumferential failure mode (Figure 9) while the open symbol represents the interfacial failure mode (Figure 10). As shown in Figure 8, the welds made by the three weld tools have similar trends of failure loads as functions of various processing parameters. The maximum failure loads of welds made by the three weld tools, marked by dashed circles, occur at similar processing parameters. Furthermore, the welds made by the three weld tools under identical processing conditions have similar failure modes as well. Only the welds made by T1 and T2 tools show the interfacial failure mode at relatively low processing parameters. Otherwise, most of the welds show the circumferential failure mode. Notably, the failure behaviors shown in Figure 8 have similar trends as those reported in Tozaki et al. [10]. The failure modes were then studied by pictures and optical micrographs of tested welds.

Figure 9 shows partially failed and failed FSSWs observed in the circumferential failure mode. As shown in Figure 9a, a crack, marked as F2, appears to emanate near the left original crack tip of the weld and then propagate along the nugget circumference. Notably, F2 is located near the boundary of TMAZ. It is interesting that Tozaki et al. [2,10] showed the FSSWs failed in similar failure mode have different failure process. The crack grows along the hook curve to the hook tip and then propagates through the upper surface. When the load continues to increase, the upper sheet is torn off with a portion of the nugget at F1, as shown in Figure 9b. The experimental observations suggest that crack F2 appears to be the dominant crack that causes the failure of the specimen.

It is interesting that the failure load of FSSWs correlates well with the hook tip distance and effective sheet thickness. The main reason is that the failure initiates from the interfacial hook, grows upward along the interfacial curve to the hook tip. Finally, the failure propagates through the upper sheet. However, this concept may not be available for the typical failed FSSW in Figure 9 since the failure occurs a bit away from the hook. Therefore, the size of TMAZ is selected as the feature to correlate the failure load.

Figure 10 shows partially failed and failed FSSWs observed in the interfacial failure mode. As shown in Figure 10a, both cracks, marked as F1 and F2, apparently emanate from the right and left original crack tips of the weld, respectively, and then propagate through the interfacial plane. Figure 10b shows the cross section along A-A plane of the nugget. A small remaining ligament (marked by an ellipse) existing near the nugget center confirms the failure process of cracks F1 and F2. When the load continues to increase, the upper sheet is sheared off with the upper half of the nugget. Figure 10c reveals the fracture surface of interfacial failure due to shear. The experimental observations suggest that both cracks F1 and F2 apparently are the dominant cracks that cause the failure of the specimen.

### 5.3. Micro-Hardness of FSSWs

**Figure 11:**
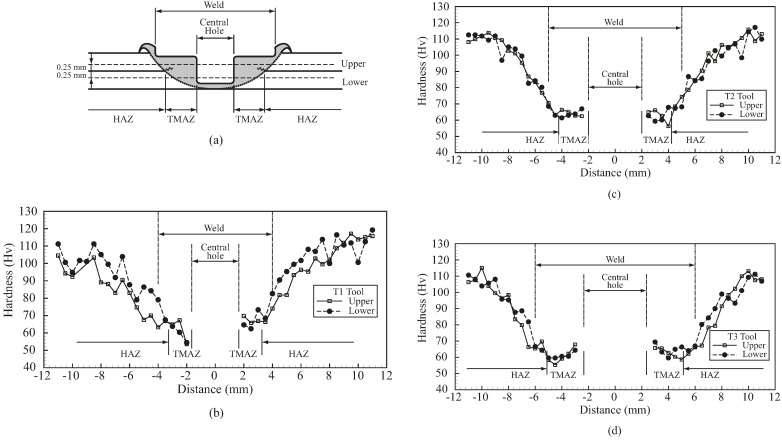
(**a**) A schematic plot of two horizontal lines for micro-indentations on the cross section of a FSSW; (**b**–**d**) Micro-hardness distributions for FSSWs made by T1, T2 and T3 tools, respectively.

Micro-indentation testing was conducted to obtain micro-hardness distributions through the cross sections of welds. As shown in Figure 11a, the two dashed lines show the indentation locations during the test. The heat affected zone (HAZ) and TMAZ ranges are also defined here. Figure 11b–d show that the welds made by the three weld tools under identical processing conditions have very similar micro-hardness distributions. Notably, the TMAZ ranges marked in the figures are measured from those in Figure 6. The micro-hardness in the HAZ gradually decreases to a minimum value near the TMAZ boundary and then slightly increases near the central hole. These figures reveal that the TMAZ and HAZ ranges of welds made under identical processing conditions are mainly determined by tool dimensions. Notably, the micro-hardness of base metal (BM) is nearly 120 Hv, i.e., higher than those of both HAZ and TMAZ. Sato et al. [11] indicated that the softening of the material in HAZ and TMAZ is possibly due to the significant reduction of dislocation density and precipitate distribution.

### 5.4. Temperature during FSSW

**Figure 12:**
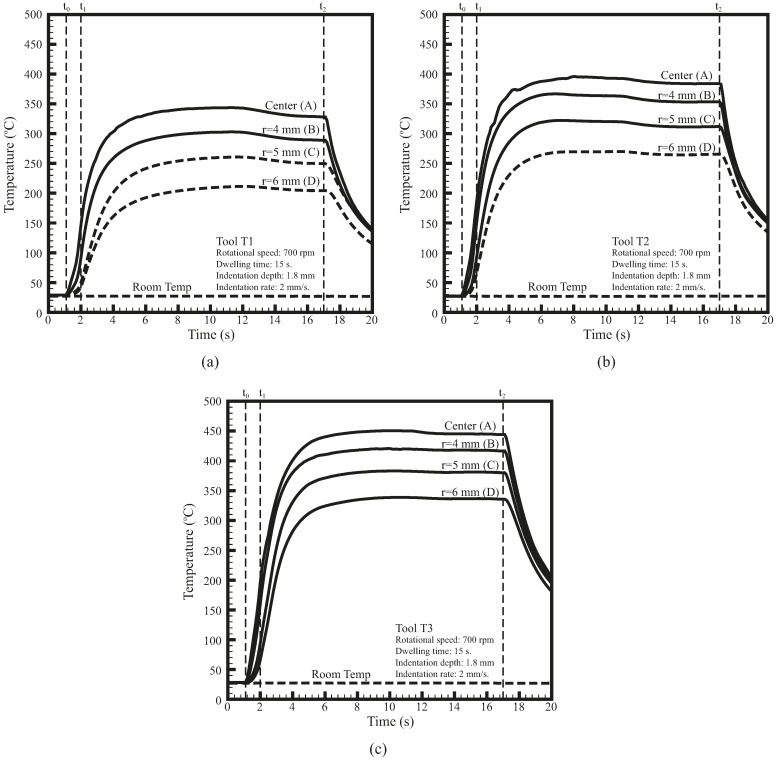
Temperature as functions of time at locations A, B, C, and D (Figure 4) for welds made by (**a**) T1; (**b**) T2 and (**c**) T3 tools.

After comparing weld quality, the performances of the three weld tools were further evaluated by temperature during FSSW. Figure 12 shows the temperatures as functions of time at locations A, B, C, and D (Figure 4) for welds made by the three weld tools. The $t_{0}$ represents the time when the weld tool initially contacted the specimen, $t_{1}$ represents the time when the weld tool was indented to a depth of 1.8 mm, and $t_{2}$ represents the time when the weld tool was extracted from the specimen. The thick dashed lines represent the temperatures measured outside the tool shoulder. As shown in Figure 12, the temperature histories for the three weld tools are quite similar. When the probe is indented into the specimen (from $t_{0}$ to $t_{1}$), the temperature below the nugget at location A rises rapidly. The temperatures below the nugget at locations B, C, and D then rise rapidly as the tool shoulder is gradually indented into the specimen. Shortly after the weld tool is indented to a depth of 1.8 mm, the temperatures at locations A, B, C, and D gradually increase to the maximum values and then slightly decrease. The temperatures finally tend to stabilize until the weld tool is extracted from the specimen. Notably, the temperature data shown in Figure 12 exhibit similar trends as those observed at the contact surface between the tool and weld in Gerlich et al. [12].

Figure 12 shows that the average rates of temperature change $\dot{T}$ at the center of the three weld tools (location A) from $t_{0}$ to $t_{1}$ are 182, 219, and 249 °C/s, respectively. The relative deviations in $\dot{T}$, $\delta_{\dot{T}}$ ($=\left(\dot{T}-{\dot{T}}_{T2}\right)/{\dot{T}}_{T2}$), in T1 and T3 tools are −16.8% and 13.7%, respectively. Additionally, the maximum temperatures $T_{max}$ at the center of the three weld tools (location A) are 344, 401, and 405 °C, respectively. The relative deviations in $T_{max}$, $\delta_{T}$ ($=\left(T_{max}-T_{\text{max}\_T2}\right)/T_{\text{max}\_T2}$), in T1 and T3 tools are −14.2% and 12.5%, respectively. It is interesting that the relative deviations in α, $\delta_{\alpha }$, in T1 and T3 tools are −13% and 14%, respectively (Table 1). These values are very similar to those of the $\delta_{\dot{T}}$ and $\delta_{T}$ for T1 and T3 tools. This indicates that the parameter α correlates not only with frictional heat generation rate density but also with welding temperature.

In Figure 4, the thermocouples are placed at 0.4 mm below the specimen in the fixture. The shortest distance from the probe tip, the maximum heat generated region, to the thermal couple is 0.6 mm. The thermal conductivities of the steel fixture and aluminum sheets degrade the welding temperatures obtained in Figure 12. On the other hand, the thermocouples in Gerlich et al. [12] are placed at only 0.2 mm from the probe tip. Therefore, the maximum temperatures obtained in Figure 12 are much less than those in Gerlich et al. [12].

In this study, the main objective for measuring the welding temperature under the specimen (Figure 12) is to obtain their general trends and confirm the concept of heat generate rate in our thermal model, not to obtain the accurate values of the maximum welding temperature inside the weld. From this viewpoint, the thermal conductivity of the fixture or the distance between the thermal couple and probe tip may not be the critical points in this study.

## 6. Discussion

For the welds made by the three weld tools, although their general trends of failure loads are similar, their maximum failure loads occur at slightly different processing conditions (Figure 8). The maximum failure loads of T1 tool occur at relatively high rotational speed or long dwelling time, while those of T3 tool occur at relatively low rotational speed or short dwelling time. Similarly, the failure mode of T1 tool changes from circumferential failure to interfacial failure at relatively high processing parameters whereas that of T3 tool keeps in circumferential mode even at relatively low processing parameters. In addition, T1 tool has lower welding temperature but T3 tool has higher welding temperature compared to T2 tool (Figure 12). The slight differences in failure load, failure mode, and welding temperature are possibly due to the differences in parameters α and β (Table 1).

Although the combined approach intend to provide new weld tools with similar α and β values, the α and β values of T1 and T3 tools are still not identical to those of T2 tool (Table 1). T1 tool has lower α value but slightly higher β value than T2 tool. According to the aforementioned results, its lower α value appears to suppress the effects of its higher β value and affects its failure load, failure mode, and welding temperature. This indicates that to provide sufficient welding temperature for obtaining satisfactory failure load and failure mode, T1 tool requires slightly higher processing parameters (Figure 8). On the other hand, T3 tool has higher α value but slightly lower β value than T2 tool. Similarly, its higher α value appears to compensate for the effects of its lower β value. Therefore, T3 tool requires relatively low processing parameters obtain satisfactory failure load and failure mode (Figure 8).

For the welds in lap-shear specimens, the annular bonding area in the TMAZ appears to be the main factor of their failure loads (Figure 5). The annular bonding area is directly related to the radii of the shoulder and probe (Figure 1). For the three weld tools in Table 1, the annular areas of their shoulders are 13.28, 21.0, and 30.48 mm^2^, respectively, which are directly proportional to their tool sizes. This result confirms the proportional relationships between tool size, annular bonding area, and failure load (Figure 8).

For each weld tool, the TMAZ size of 15 s is very similar to that of 7 s (Figure 6 and Figure 7). The remaining processing parameters of these welds are identical. In general, the size and shape of TMAZ are strongly affected by both thermal and mechanical behaviors of the weld tool. In Figure 12, the welding temperatures increase rapidly at the beginning and then become stable as the dwelling time is greater than 5 s. The stable of welding temperature is due to the balance of heat generation and heat transfer in the process. In addition, identical rotational speed and indentation depth may result in similar plastic deformation in the TMAZ. This indicates that the TMAZs of welds made under dwelling time of 7 s and 15 s may experience similar welding temperature and plastic deformation. Thus, they have similar TMAZ sizes and shapes.

**Figure 13:**
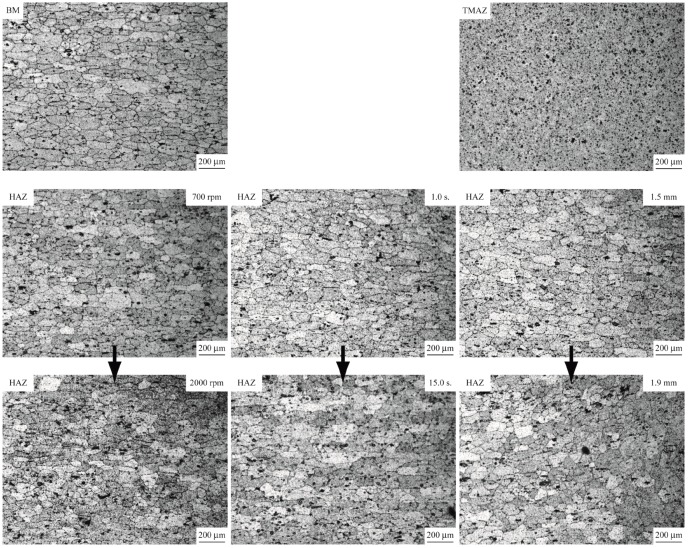
Optical micrographs of BM, TMAZ, and HAZ in welds made by T2 tool at processing parameters listed in Table 3.

It is interesting that not only the size and shape of TMAZ but also the microstructure of HAZ are strongly affected by the processing parameters. Figure 13 shows the micrographs of BM, TMAZ, and HAZ for welds made by T2 tool at the processing parameters listed in Table 3. The BM with medium grain size and the TMAZ with fine grain size are taken as references. In Figure 13, as the rotational speed and dwelling time increase to 2000 rpm and 15 s, respectively, the grain size in the HAZ increases probably due to more heat generation. The amount of large particles (dark spots) precipitated in the HAZ increases as well. Based on the Hall–Petch relationship, the grain size is inversely proportional to the material strength. Sato et al. [11] indicated that the reduction of small particles (<30 nm), caused by precipitation of large particles, softens the material in HAZ. Also, the circumferential failure in general occurs near the boundary between the HAZ and TMAZ (Figure 9). Thus, the failure loads of the welds made at 2000 rpm and 15 s are relatively low (Figure 7). On the other hand, as the indentation depth increases to 1.9 mm, the grain size in the HAZ slightly changes. This indicates that the indentation depth appears to contribute more on plastic deformation rather than heat generation.

Generally speaking, under identical processing conditions, the TMAZ and HAZ sizes of the welds made by T1, T2, and T3 tools are proportional to tool size (Figure 7 and Figure 11). Their TMAZ profiles are quite similar as well (Figure 6). Their failure loads are approximately proportional to tool size and their failure modes are also similar (Figure 8). The above results reveal that although the weld radii are different, the qualities of welds made by the three weld tools, including microstructure, micro-hardness distribution, failure load and failure mode, are quite similar under identical processing conditions. Notably, the three weld tools, designed based on the thermal-mechanical model and combined approach, have similar α and β values (Table 1). As mentioned earlier, parameters α and β, which correlate with frictional heat generation rate density and stirring power density, respectively, substantially affect weld quality. This indicates that weld tools with similar α and β values can easily make welds of various sizes but similar qualities under identical processing conditions.

## 7. Conclusions

A novel tool design approach based on the approximate thermal–mechanical model for FSSW and the combined approach was developed to design tools with comparable performances for welds sizes of 8, 10, and 12 mm. The performances of the three weld tools were then evaluated based on experimental approaches. Effects of shoulder radius, probe radius, probe length, and sheet thickness on welding performance were discussed based on the thermal–mechanical model. The experiments confirmed the following:

1. Given identical processing conditions, the failure loads of welds made by the three weld tools were approximately proportional to tool size; the failure modes in general were identical; the microstructures and micro-hardness distributions were also similar.
2. The temperature change rates and maximum temperatures of the three weld tools correlated well with their frictional heat generation rate densities.
3. The thermal–mechanical model indicated that, for a given shoulder radius, welding performance increases as probe radius or length increases but decreases as sheet thickness increases.

In summary, the experimental results confirmed that the three weld tools sufficiently met all design objectives, which indicates that the proposed design approach is a simple and feasible guideline for tool design.

## Figures and Tables

**Table 1:** Geometric parameters, parameters *α* and *β*, and relative deviations $\delta_{\alpha }$ and $\delta_{\beta }$ of weld tools developed by the thermal-mechanical model (Unit: mm).

Tool	*t*	*r_s_*	*r_p_*	*L_p_*	*α*	*β*	*δ_α_*	*δ_β_*
T2	1.0	5.0	2.0	1.6	2.50	0.29	0	0
T1a	1.0	4.0	1.9	1.6	2.50	0.44	0	0.49
T3a	1.0	6.0	1.86	1.6	2.50	0.17	0	−0.43
T1b	1.0	4.0	1.6	1.6	2.12	0.29	−0.15	0
T3b	1.0	6.0	2.4	1.6	2.88	0.29	0.15	0
T1	1.0	4.0	1.65	1.6	2.17	0.32	−0.13	0.07
T3	1.0	6.0	2.35	1.6	2.84	0.28	0.14	−0.05

**Table 2:** Chemical compositions (wt. %) of aluminum 6061-T6 sheets.

	Si	Fe	Cu	Mn	Mg	Cr	Zn	Ti	Al
Minimum	0.4	-	0.15	-	0.8	0.04	-	-	
Maximum	0.8	0.7	0.4	0.15	1.2	0.35	0.25	0.15	remainder

**Table 3:** Processing parameters of the FSSWs shown in Figure 6.

Figure	Rotational Speed (rpm)	Indentation Depth (mm)	Dwelling Time (s)	Indentation Rate (mm/s)
6a	700 2000	1.8	7	2
6b	700	1.5 1.9	7	2
6c	700	1.8	1 15	2
